# Guidance for Residents Addressing Copper Problems in Drinking Water: Opportunities and Challenges

**DOI:** 10.1021/acsestwater.4c00447

**Published:** 2024-08-12

**Authors:** Rebecca Kriss, Marc A. Edwards

**Affiliations:** Civil and Environmental Engineering, Virginia Tech, 418 Durham Hall, Blacksburg, Virginia 24061, United States

**Keywords:** drinking water, copper corrosion, cuprosolvency, corrosion control treatment, residential guidance

## Abstract

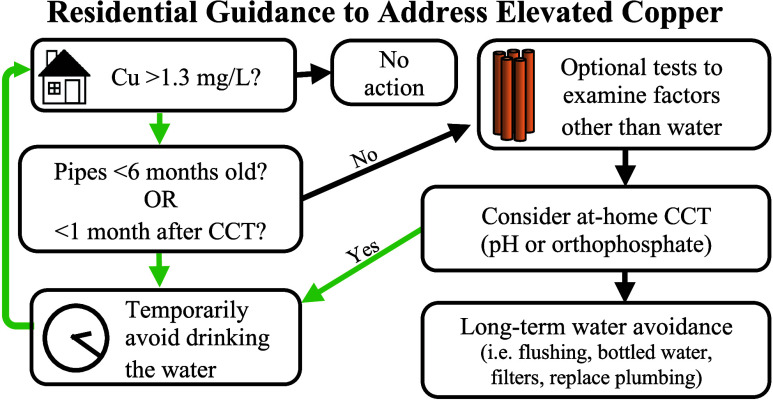

Residents and their pets may experience aesthetic or health concerns resulting from elevated copper in their drinking water. The United States Environmental Protection Agency Lead and Copper Rule focuses on addressing systemwide corrosion issues, but gaps in the rule leave some municipal water consumers and residents with private well water vulnerable to high cuprosolvency. We developed guidance to aid residents in understanding, detecting, and addressing cuprosolvency issues in their drinking water. Three types of at-home test kits for copper and one for pH were determined to be accurate enough (*R*^2^ > 0.9 (lab, based on average values from *n* = 5 replicates each) and >0.7 (field)) to detect concerns related to high cuprosolvency and inform selection of intervention options. Case study results indicate that, although water treatments such as increasing pH on-site may be effective, long-term treatment (>36 weeks or permanently) may be needed to maintain reductions in cuprosolvency. A decision tree is provided to help residents and citizen scientists navigate these concerns for both public water systems and private wells.

## Abstract 2

This work addresses gaps in the LCR by developing guidance to help residents detect and address elevated copper in their drinking water.

## 1 Introduction

Elevated copper in drinking water results from corrosion of copper plumbing. High cuprosolvency, i.e., copper release to water, is expected in new plumbing and may persist for days, months, years, or decades until low-solubility mineral coatings, or “protective scales,” form inside pipes. Whether these coatings form, and when, depends on water chemistry.^1−7^ Residents with cuprosolvency issues may experience aesthetic concerns, like blue water and fixture staining, and health effects, like acute gastrointestinal distress, liver and kidney issues, and more serious effects for sensitive populations (e.g., Wilson’s disease) and some pets.^1,8−10^ Other manifestations of copper corrosion, such as pitting, wall thinning, and erosion corrosion, can cause leaks and pipe failure, but are not the focus of this paper.^11^ To address systemwide cuprosolvency issues, the United States Environmental Protection Agency (USEPA) Lead and Copper Rule (LCR) set a 1.3 mg/L action level (AL) for copper in municipal systems. Copper concentrations for more than 90% of homes in LCR sampling must be below this AL, or utilities must notify the public and implement corrosion control treatment (CCT).^2,6,9,12^ In some cases, even when utilities implement CCT, elevated cuprosolvency may persist indefinitely, as was observed for a utility adding polyphosphate corrosion inhibitors.^13^

The LCR may leave some residents unprotected from elevated copper in drinking water. Because the LCR uses a 90th percentile AL, up to 10% of residents may have elevated copper above the AL. In addition, homes sampled generally do not represent the worst cuprosolvency issues because the rule prioritizes sampling older homes at highest risk of lead, rather than homes with new copper pipes which tend to have higher copper.^2,6^ Therefore, LCR sampling likely underestimates cuprosolvency issues for some new homes, and the extent of this underestimation keeps increasing as homes in the LCR sampling pool age. Finally, because the LCR regulates water quality of homes in municipal systems, elevated copper exposure from drinking water in private wells and nonresidential buildings are not addressed.^1,2,12,14^ These gaps leave all private well owners and many residents and nonresidential building owners with municipal water at risk of cuprosolvency issues in their drinking water.^6,8,15^

Consumer-centric tools and guidance are needed to help address elevated cuprosolvency. Guidance was previously developed to help utilities address cuprosolvency based on key water quality parameters such as pH, alkalinity, and orthophosphate dose.^16−18^ That guidance serves as a starting point, but needs to be modified to consider sampling methods and treatments appropriate for residential use. Some at-home tests for lead and chlorine in drinking water were relatively accurate and appropriate for citizen science use,^19,20^ suggesting that similar tests may be promising for measuring copper and water quality parameters needed to guide consumer-centric strategies.

Ideally, a hierarchy of treatment strategies could be proposed with a preference for effective, less costly strategies that work quickly (Table 1). One-time, site-specific treatments that could facilitate permanent formation of a low-solubility (i.e., protective) scale would be ideal for residential applications, such as granular activated carbon (GAC) removal of organic matter, suggested by Arnold et al., or anion exchange treatment.^6^ However, researchers have noted difficulties applying laboratory cuprosolvency results to the field, indicating a need for further study to determine if and when they would be successful in real systems.^1,7^ If inexpensive one-time treatments do not work, longer-term or permanent water chemistry adjustments (i.e., dosing phosphate, raising pH) may be needed to reduce cuprosolvency. However, if these treatments pose too much long-term expense and are too complicated (installation, maintenance, reagents), residents may prefer other steps like expensive one-time replumbing with plastic pipe, flushing, use of NSF 53 certified filters, or drinking bottled water.

**Table 1:** Hierarchy of Potential Residential Interventions to Address Copper in Drinking Water^a^^,^^b^^,^^c^

	**confidence of resolution**				
**intervention**	**consumption and cooking**	**household water**	**initial capital cost**	**ongoing maintenance**	**additional considerations**
replumb with plastic or stainless steel	certain		$$$	0	high cost and construction in home
bottled water	certain	no effect	$	$$	no effect on fixture staining or green hair
point of use tap or pitcher filter^d^	likely	no effect	$	$	at present no tap or pitcher filters are NSF/ANSI 53 certified for copper removal
point of use reverse osmosis^d^	certain	no effect	$$	$	NSF/ANSI 58 certified for copper removal
whole house orthophosphate addition	high		$$	$	active chemical dosing likely required.
whole house pH adjustment	high, dependent on water chemistry		$	$ or $$	active chemical dosing of caustic, or passive limestone/soda ash contactors
whole house GAC, reverse osmosis, ion exchange	unproven/unknown, dependent on water chemistry		$$	$$	more research is needed

a GAC refers to Granular Activated Carbon filtration.

b Confidence of resolution hierarchy is as follows: Certain > High > Likely.

c Approximate costs are as follows: $ indicates less than $50 initial capital cost or maintenance per year. $$ indicates $300–$1000 initial capital cost or maintenance per year. $$$ indicates more than $1000 initial capital cost or maintenance per year.

d NSF/ANSI Certified point of use filters^21^ and reverse osmosis treatments.^22^

This study aims to develop preliminary residential guidance to detect, diagnose, and remediate elevated cuprosolvency in drinking water. A decision framework was adapted from prior research to utilize testing and intervention strategies appropriate for individual residences. This framework could be used by individual residents or adapted by water authorities (i.e., utilities, health departments, cooperative extension programs) to provide location-specific guidance accounting for concerns/conditions in specific water systems or water sources. The laboratory and field accuracy of inexpensive at-home test kits were evaluated for providing data to navigate the framework. We also demonstrated the efficacy of potential interventions in both lab and field studies and use of a simple diagnostic cuprosolvency test to rule out grounding or pipe defects. Finally, we illustrate use of the evolving framework via case studies with two residents attempting to remediate cuprosolvency issues.

## 2 Materials and Methods

### 2.1 At-Home Test Kit Testing

At-home test kits for copper and pH were tested according to manufacturer instructions to determine their accuracy in the laboratory (1 participant, blind sampling, *n* = 5 tests per condition) across a range of pH and copper concentrations. Test kits included one from each of three categories: color tile strips and liquid-based color change kits for pH and copper and a low-cost field colorimeter for copper (Figure S1). Laboratory test waters were comprised of deionized water adjusted to target pH values (6.0 to 8.8) or with cupric nitrate (0 to 5 mg/L copper) and performed within 1 h of copper addition. Field accuracy of test kits was determined via residential sampling performed by Resident B.

### 2.2 Residential Water Sampling and Testing

Residential testing was performed in the homes of two residents. Both residents receive their water from the same utility and recently replumbed their homes with copper pipe (Resident A in 2017, Resident B from 2014–2017).

Resident A collected water samples (250 mL) at two locations (kitchen tap and hose bib) in their home. For comparison a tap in a next-door building with copper pipes >40 years old was sampled. One sample was collected after flushing approximately 5 gallons of water (5 min at kitchen tap), another was collected immediately upon opening the tap after a prescribed stagnation time (4 h during the day, 10 h at night, up to 24 h in subsequent tests), and a third after 20 s of flushing poststagnation. Because the next-door building has daytime occupants, daytime stagnation goals could not be ensured. Resident A determined electric current on pipes via a method suggested by a technical expert at a major multimeter manufacturer, whereby current was measured between the hose bib or water meter and a grounded pipe segment in the yard away from the plumbing. Onsite cuprosolvency tests were performed using 8.5-in. copper pipe segments (*n* = 5) filled with water collected from the residence after flushing 5-gallons of water. Water was collected from these ungrounded pipe segments (i.e., with no current) for copper analysis after the 10-h overnight stagnation period, for comparison to samples from home plumbing potentially affected by grounding.

In-home sampling was performed by Resident B as reported in Wait et al.^23^ Resident B collected three samples (250 mL) weekly, each from a tap in their home and in a guest house on their property, from March of 2021 to May of 2022. Samples were collected after a 5 s flush to obtain water from copper plumbing and included samples collected immediately upon opening the tap, after 5 min of flushing, and after water was stagnant for 24 h. Samples were analyzed in the field using citizen science testing with at-home tests and with a calibrated field pH probe. Laboratory analysis was used to confirm copper concentrations. A whole-house treatment system using soda-ash pH adjustment was installed by Resident B and periodically utilized to attempt to reduce cuprosolvency.^23^

### 2.3 Water Treatment Testing

Laboratory cuprosolvency tests examined the corrosivity of three source waters from the consumers’ utility: a surface water and two well waters, and simulated the effects of several water treatments using Resident B’s water. Tests followed similar protocols to that of Kriss et al., but were performed at ambient temperature.^17,18^ In brief, experiments used 8.5-in. copper pipe segments stoppered at one end with silicone stoppers (*n* = 5 per condition), with water changes 3 times per week via a dump and fill protocol for 37 weeks. Weekly composite samples and monthly individual pipe samples were analyzed for copper. The utility indicated that Resident B likely received water from multiple sources, so one surface and two nearby well source waters were tested. Additional water conditions tested include reverse osmosis treated water, and water sent by the resident and treated using soda-ash pH adjustment (initial pH 7.5, pH 8.8 and pH 9.5), GAC filtration, and filtration using an anion exchange resin.

### 2.4 Analytical Methods

Standard methods were used to analyze base water quality parameters. Samples were analyzed for copper using atomic absorption spectroscopy (AAS, PerkinElmer 5100 PC AAS) via method 3111B or inductively coupled plasma-mass spectrometry (ICP-MS; Thermo Scientific iCAP RQ ICP-MS) via method 3125 B.^24^ ICP-MS analysis was also used to determine concentrations of other metals, phosphorus, silica, and sulfate. Analyses using AAS and ICP-MS were performed after acidification of samples with 2% nitric acid for at least 16 h after confirming there were no visual particulates/solids.

## 3 Results and Discussion

### 3.1 Residential Guidance Framework

Gaps in the LCR leave many residents vulnerable to persistent elevated copper in drinking water, demonstrating the need for guidance to help them address cuprosolvency issues. Ideally, water coming into residents’ homes would be noncorrosive to new copper pipes. However, utilities are not required to implement CCT for copper in new homes under the LCR, and many residents/building owners are solely responsible for their water quality (e.g., private wells).^2,6,8,9,12^

**Figure 1:**
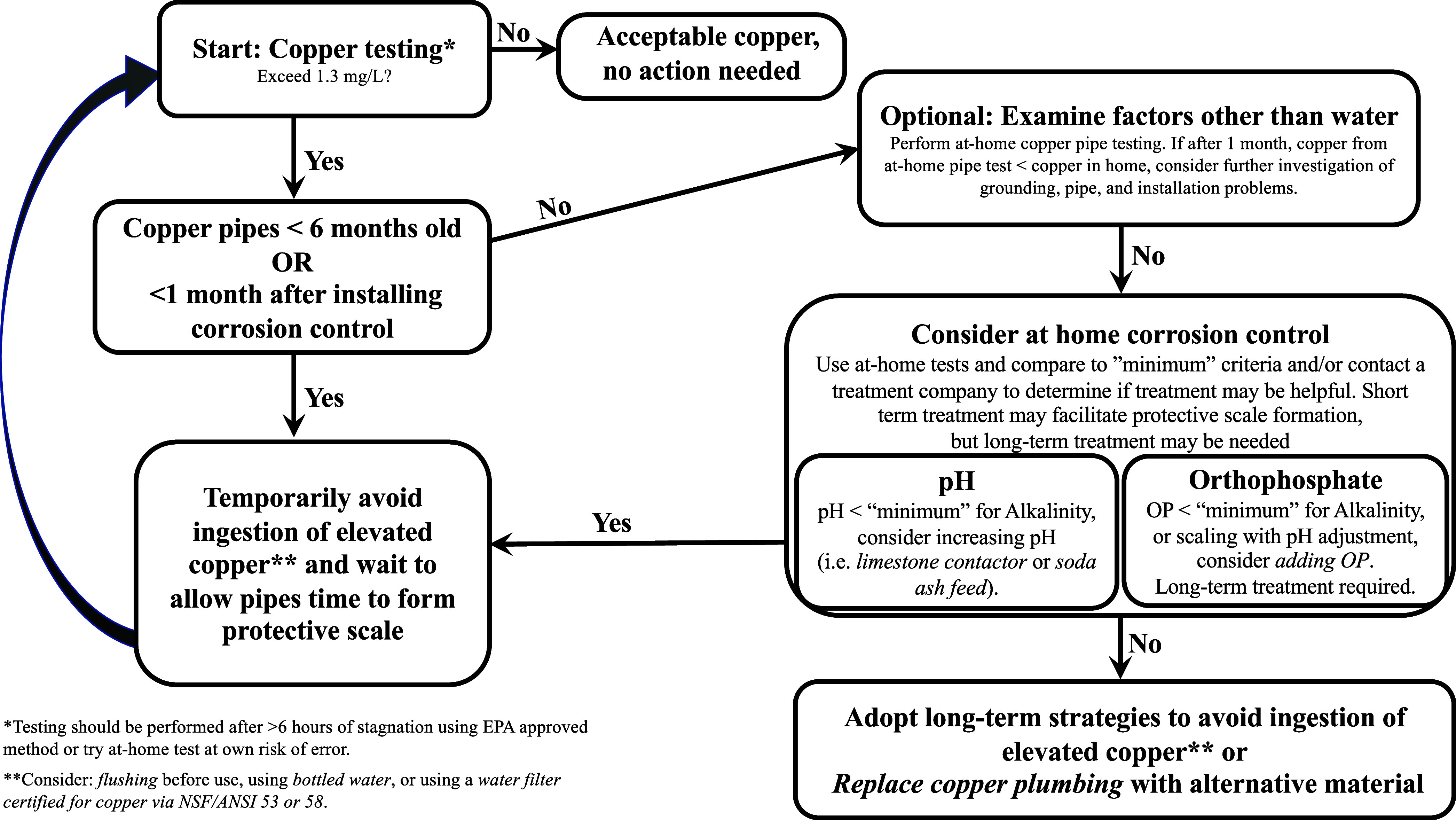
Resident centric decision-making framework to address cuprosolvency issues. “Minimum” criteria for potential corrosion control were determined in previous studies.^17,18^

A guidance framework was developed to mitigate risks and help residents detect and address cuprosolvency issues (Figure 1). In this approach, residents would initially determine whether they have elevated copper in their drinking water. If their copper pipes are relatively new, they are advised to wait and temporarily avoid ingesting the water until pipes are >6 months old, giving time for a protective scale to form. If high cuprosolvency persists for >6 months, residents may wish to perform simple at-home cuprosolvency tests to rule out factors often blamed for high cuprosolvency such as bad plumbing, flux, or improper grounding, even though they are rarely the cause of such problems in our experience.^25,26^ Residents can also consider adjusting water quality through whole-house treatments to facilitate formation of protective scales. This includes using relatively simple off-the-shelf treatment systems with common CCT methods such as pH adjustment (i.e., via limestone contactor or adding soda ash) or adding orthophosphate corrosion inhibitors, with treatment targets guided by criteria from previous work.^16−18^ Additional treatments, such as GAC filtration, might reduce cuprosolvency in some cases, but are less likely to be successful.^6^ Finally, residents can take steps to avoid ingesting water with elevated copper (flushing, NSF 53 certified filter, bottled water) while waiting for protective scales to form, if they prefer these over other interventions, or if other approaches are unsuccessful. Although costly, replacing copper pipes with stainless steel or plastic pipes can be considered. This approach may be helpful for individual residents, building managers, and proactive utilities when cuprosolvency concerns can be addressed on small-scale bases and are not widespread enough to warrant utility-wide CCT.

### 3.2 Accuracy of At-Home Copper and pH Test Kits

**Figure 2:**
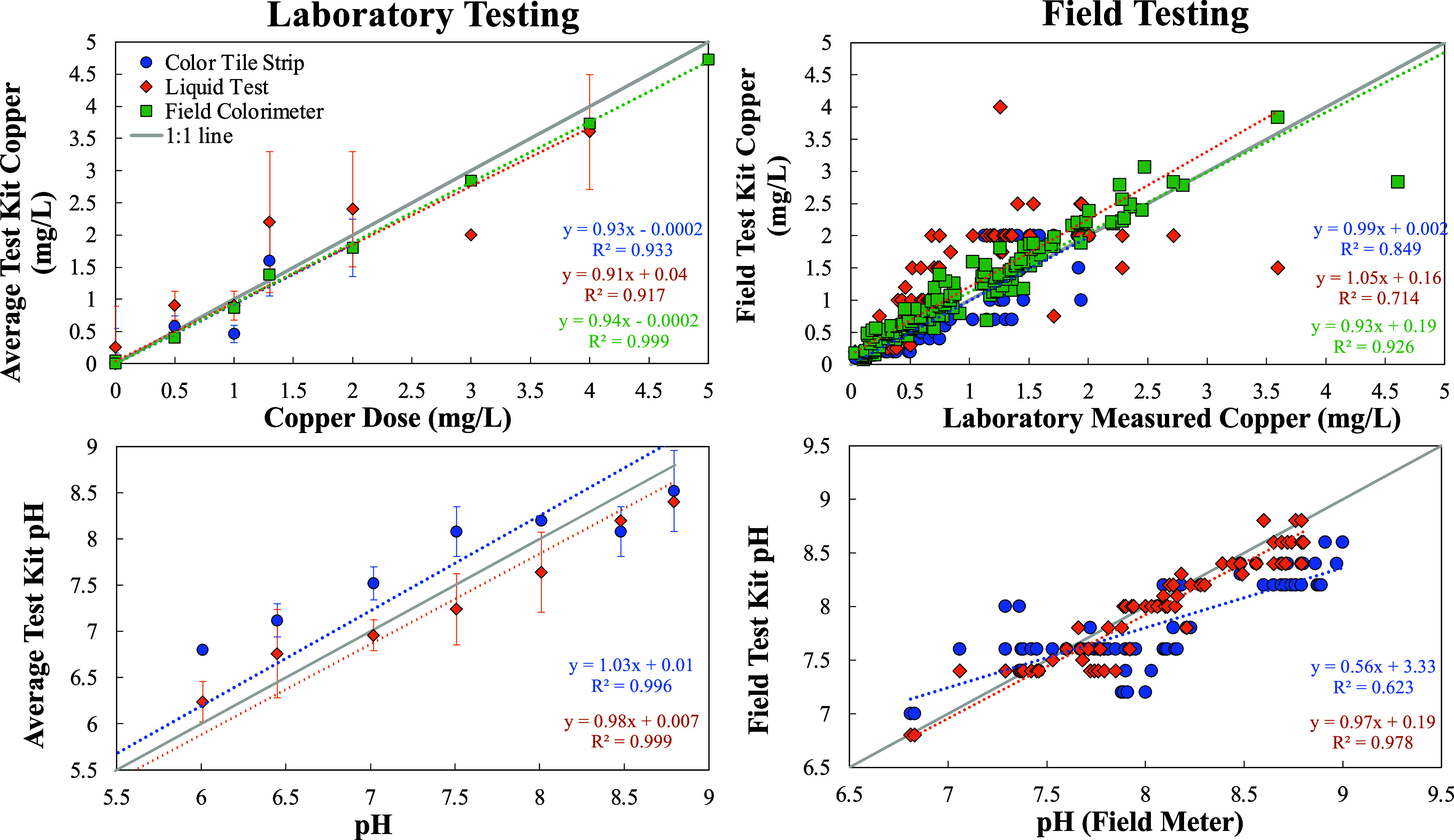
Laboratory and field accuracy of at-home pH and copper tests. Laboratory and field accuracy of pH and copper at-home test kits (color tile, liquid, field colorimeter). Laboratory results were determined using a blind testing approach (based on average values of *n* = 5 replicates), and error bars representing one standard deviation. Field results were measured by Resident B over the course of 1.5 years of in-home sampling. Actual values were determined via ICP-MS/MS laboratory analysis (copper) and a calibrated pH meter.

While consumers could pay for costly sampling by certified laboratories, inexpensive consumer-centric tools could help residents determine if they have elevated copper and identify water quality parameters that could affect treatment recommendations. Many at-home tests for parameters like copper and pH are available for drinking water, pool, and aquarium uses. Three at-home tests for copper and two for pH, representing 3 general categories of test, were evaluated in laboratory and field trials to determine their accuracy and general usability (Figure 2). Tests included color tile strips ($14.50 for 50 strips) and liquid tests ($9–12 for 90 tests) for both parameters and a field colorimeter for copper detection ($68 plus $18 for 25 tests or $50 for 100 tests). All kits utilize color changes to indicate copper concentrations or pH values, but the field colorimeter gives a more precise digital readout.

Results indicate that the field colorimeter is the most accurate option for relatively low-cost detection of copper in drinking water. The field colorimeter exhibited good accuracy in both the laboratory and field (*R*^2^ > 0.93), with relatively small variability (laboratory relative standard deviations (L-RSDs) 1–15%) and no false negative results (i.e., the kit falsely indicated copper below the AL), making it the best option for copper detection. The liquid test was the least accurate (laboratory *R*^2^ = 0.92, field *R*^2^ = 0.71), and exhibited more variability (L-RSDs 0–50%), but still low false negative rates of 5%. Finally, even though the color tile strip was generally accurate in the laboratory and field (laboratory *R*^2^ = 0.93, field *R*^2^ = 0.85), it exhibited substantial variability (L-RSDs 25–35%) and the highest false negative rate of 30%, making it the least reliable choice for detecting copper. These results demonstrate the relatively inexpensive field colorimeter is accurate enough to serve as an initial screening tool to detect copper above the AL and potentially monitor treatment progress.

Laboratory results indicate that liquid and color tile strip pH tests are relatively accurate (*R*^2^ > 0.99 and low standard deviations < 7%). However, the liquid test (*R*^2^ = 0.978) was more accurate in the field than the color tile strip (*R*^2^ = 0.623). Therefore, the liquid test is recommended for residential use and could be used with the framework to indicate whether pH CCT could be a viable treatment strategy. Residents should consider if more sophisticated tests are needed prior to investing in whole-house treatment strategies.

### 3.3 Citizen Science Case Studies

This section illustrates the experiences of two residents navigating the guidance framework while trying to address cuprosolvency problems in their homes. Both residents receive water from the same municipal water system, which has not reported systemwide cuprosolvency problems since at least 2013 (i.e., current 90th percentile copper below 0.31 mg/L).^27^ Despite this, these residents experienced prolonged elevated cuprosolvency after installing new copper pipe during home renovations. Other residential buildings on the same water system reported elevated copper in 2017, suggesting that more widespread issues may be present than revealed during LCR sampling.^28^

#### 3.3.1 Resident A Experience Investigating Non-Water Factors

Resident A completed installation of new copper pipes in 2017. Two years later, their dog became deathly ill with symptoms later determined to be consistent with heavy metal poisoning, and seemingly made a complete recovery after switching from tap to bottled water.^29^ By September of 2020 the resident was notified of elevated copper (1.53 mg/L) in their water, which was found through routine LCR sampling. Even higher copper concentrations (2.45 mg/L) were found in follow-up sampling.^30^ This represents a case where LCR sampling detected cuprosolvency problems. However, because the utility’s 90th percentile copper concentration was less than the AL, no CCT or utility action was required (i.e., up to 10% of homes sampled can exceed the AL).

Authorities initially blamed high current due to improper grounding for this resident’s elevated cuprosolvency. The current was allegedly so high, in November of 2020, the resident was advised to leave the premises due to shock hazard.^29,30^ In the next few weeks, the resident worked with local water, gas/electric, and telecom utilities to address the issue. Even after multiple utility interventions, the stray current was reduced but not fully removed from the resident’s pipes and copper levels remained well above the AL (near 3 mg/L).^29^ In December of 2020, Resident A installed a dielectric union to electrochemically disconnect their pipes from the copper service line which reduced the remaining voltage on the pipes.^30^ But elevated copper persisted in the resident’s home.

Having exhausted other avenues, the resident contacted the authors in fall of 2021 with concerns about elevated copper, still convinced it was due to a very low direct current on their pipes. We helped them measure copper in the water in their home with relatively new pipe (<5 years old). Initial results indicated much higher copper in their home (0.5–1.1 mg/L) than in a next-door building with approximately 40-year-old pipes (<0.1 mg/L), suggesting a protective scale had not yet formed in the resident’s home. Flushing the water for 5 min at the kitchen tap (∼5 gallons) was effective at reducing copper to 0.1–0.2 mg/L.

In order to address the resident’s concerns about any remaining current on their pipes, we helped the resident test the normal copper concentrations when their water was in contact with brand new pipe segments without any electrical connection, as is suggested in the decision framework (Figure 1). This revealed higher copper concentrations in the brand-new pipe segments with no current (0.98–1.31 mg/L) versus the <5-year-old grounded pipes in the home (0.50–0.96 mg/L) (Table S1), proving that the water alone could be responsible for the high copper. The resident also performed in-home sampling which yielded no consistent relationship between current and copper concentrations.

In the summer of 2022, because copper levels stayed high (1–2.4 mg/L), the resident installed a sacrificial anode in a futile attempt to address any remaining current issues. Over the course of 2 months, the resident installed, connected, disconnected, removed, and moved the anode, with no discernible effect on the copper concentrations in their water. Although this resident did initially have grounding issues, their more recent results are consistent with our experience that grounding is rarely the cause of elevated copper in water. In the end, the resident was relying on a reverse osmosis filter and considering replacing all their copper pipes. All of this demonstrates the struggles and frustrations of having individual residents navigating cuprosolvency issues without rational assistance.

#### 3.3.2 Resident B Experience Investigating Water Quality Factors and Potential Treatments

Resident B’s experience, first reported in Wait et al., and summarized in Figure S2, began with a home renovation and installation of new copper pipes from 2014–2017.^23^ In the summer of 2020, more than 3 years after the renovation, the resident suspected cuprosolvency problems after noticing blue deposits in their icemaker and a green tint to their hair. Similar hair color changes and blue deposits were observed in several instances in the community 3 years earlier.^28^ The resident used an at-home test which indicated copper concentrations above the AL (1.7 mg/L) in their water and confirmed elevated copper up to 3.88 mg/L via private laboratory testing in September and October of 2020. After hearing about the experience of Resident A, Resident B suspected that their dog, who got sick and died soon after the renovation, may have also been affected by heavy metal poisoning from elevated copper in their water.^23^

Resident B first tried to address these issues in collaboration with their local water utility. In response to local reports of elevated cuprosolvency, the water utility performed additional non-LCR testing in late 2020, including testing water in Resident B’s home.^29^ They found copper above the AL in 10 of 75 homes tested, which is above the 10% of homes required to trigger the copper action level. But only two homes still had copper above the action level after retesting using a preflushing protocol. The utility asserted that any cuprosolvency problems were an isolated occurrence, and the residents were on their own to address any cuprosolvency issues. Nonetheless, the utility continued to voluntarily cooperate with us, by providing water samples and information when requested.^23^

Resident B undertook many steps to address the cuprosolvency issues. After initially discovering elevated copper, the resident switched to drinking bottled water, and installed a point of use reverse osmosis system in their kitchen, which alleviated their green hair. The resident then began investigating and implementing whole house treatment options. First, the resident bypassed the water softener for 10 days, hoping, in vain, to cause formation of protective scales. Copper concentrations remained high at 1.59 to 3.63 mg/L after treatment, as measured by private laboratory testing. The resident then worked with a local home water treatment company to install a whole house system to raise the water pH and monitored copper concentrations with an at-home colorimeter. A soda-ash feed was chosen instead of using a limestone contactor, which has easier maintenance, due to the relatively high dissolved inorganic carbon content in the water.^23,31^

The resident had a strong applied chemistry background during a career in industry. They contacted the Virginia Tech team in February of 2021 to gain a deeper understanding of these cuprosolvency issues. We helped the resident assess several factors and assisted with laboratory copper analysis. Tests indicated that sulfate reducing bacteria, which can increase cuprosolvency, were not present.^32^ Further, tests indicated that short-term chlorination of the system via flushing or bypassing the water softener, which can sometimes promote copper oxidation and aging, was not effective at reducing copper.^33^ Testing also revealed that flushing the water for 5 min was not always sufficient to reduce copper below the action level, with concentrations up to 1.5 mg/L. Finally, we helped the resident select a target pH of 8.5 for pH adjustment with the soda ash feed. This pH was predicted to exceed the minimum criteria in the framework (Figure 1), and was therefore expected to yield a water that is nonaggressive to new copper.^16,18^

**Figure 3:**
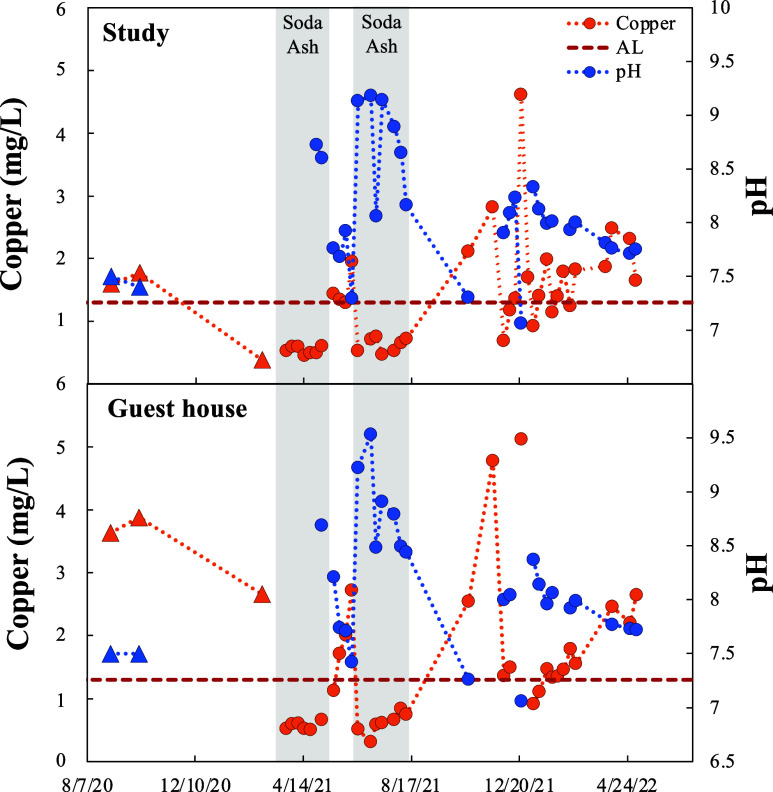
In-home testing/intervention. Copper and pH measurements from the home of Resident B at two locations. Historical data from testing in the home are represented by triangles. Circles represent first draw samples collected in this study after 24 h of stagnation.

Repeated in-home sampling revealed highly variable copper concentrations, which were frequently above the AL and up to 5.1 mg/L without any intervention (Figure 3). Continuously boosting the pH using the soda-ash feed was effective at quickly reducing cuprosolvency below the AL. However, when treatment was suspended copper concentrations increased back above the AL, indicating that a permanently protective scale did not form during either of the two 3-month periods of pH adjustment.

During the first period of treatment, it was determined that the soda-ash had a polyphosphate additive, often used to prevent scaling and reported to complex copper, which could have affected potential protective scale formation during this period.^34,35^ The resident also noted initial difficulties maintaining the target pH, having to perform frequent adjustments to the system, which may have been caused by variations in the incoming water quality. Fluctuations in the incoming pH ranged from 6.8 to 8.4 when no treatment was being used (late 2021 to early 2022). The utility then indicated that, due to the resident’s location in the distribution system, they likely received a mixture of surface and well water, which could have contributed to these variations.^23^ Using Si and Cl measurements (via ICP-MS) as a tracer indicated that about 25% of the resident’s water was from wells and 75% from surface water at the time tested. These results highlight some challenges residents may face when attempting to implement whole house treatment strategies.

**Figure 4:**
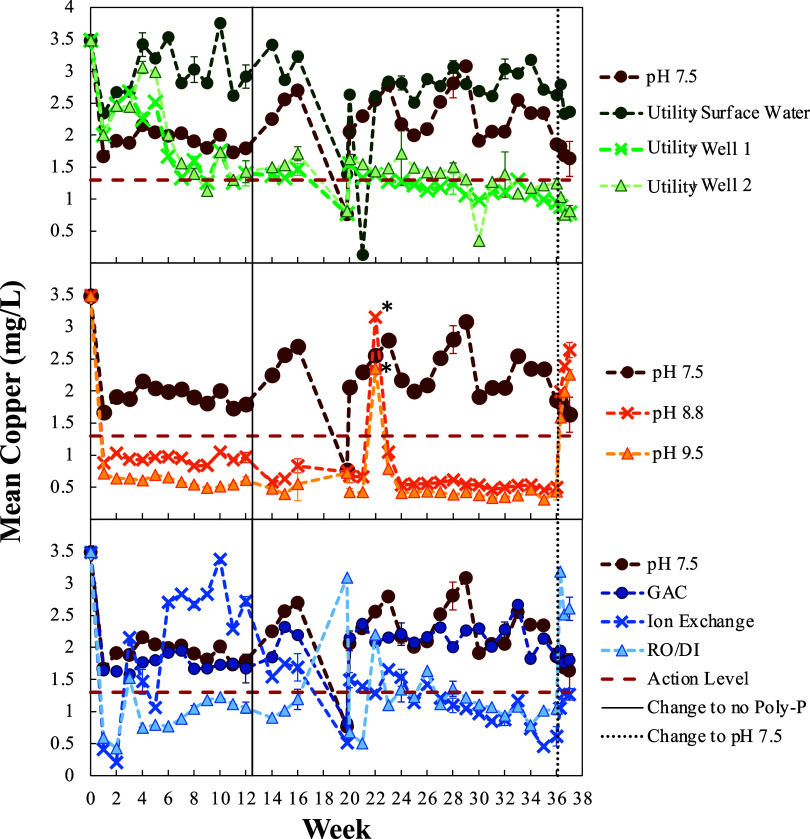
Laboratory pipe tests with interventions. Laboratory cuprosolvency tests using copper pipe (*n* = 5) and Resident B’s water (pH 7.5), Resident B’s treated water, or surface or well water from the local utility. Water treatments include filtration with granular activated carbon or anion exchange resin, reverse osmosis/deionized water, and pH adjustment using soda ash to pH 8.8 or 9.5. All waters had polyphosphate prior to week 13, except water from the local utility and RO/DI water. Polyphosphate was removed by the ion exchange treatment. All waters were changed to Resident B’s control water (pH 7.5) after week 36. Error bars represent standard deviations for full sampling events. * pH 8.8 and 9.5 waters were adjusted to pH 7.5 for week 22.

Laboratory pipe cuprosolvency studies were performed using Resident B’s water as well as water from the local utility to determine potential causes and effective treatments for the observed high cuprosolvency (Figure 4). Results indicate that Resident B’s water (pH 7.5) generally resulted in intermediate cuprosolvency between utility surface and well water sources, consistent with reports from the utility that Resident B receives a mixture of these waters. Cuprosolvency in these waters remained relatively high (>0.9 mg/L) within the 36 weeks of testing, with the resident’s water (>1.6 mg/L) and the surface water source (all but one sample >2.3 mg/L) remaining almost always above the AL. Even though the utility was not exceeding the AL and was therefore not in violation of the LCR, these results confirmed that a low solubility protective scale did not form in new pipes exposed to their water for up to 36 weeks—even without any grounding or other dubious causes of cuprosolvency. This is consistent with the elevated cuprosolvency observed in both Resident A and B’s homes even after 5 years of aging as well as elevated cuprosolvency observed in condo buildings in the community after 5–10 years of pipe aging.^28^

In order to address the cuprosolvency issues, potential treatments for the resident were evaluated at Virginia Tech using pipe cuprosolvency tests. The most effective treatment tested was the addition of soda-ash to increase the pH to pH 8.8 and 9.5, above the predicted “minimum” threshold value. Upon increasing the water pH, copper concentrations quickly fell below the AL to as low as 0.3 mg/L. However, drastic increases in cuprosolvency of 1.1–2.5 mg/L were observed when the pH was returned to the resident’s original pH 7.5 water, even after 22 or 36 weeks of treatment, indicating that a permanent protective scale had not formed. Reverse Osmosis pretreatment yielded cuprosolvency reductions from 0.3 to 1.9 mg/L in comparison to no treatment (pH 7.5 water), however copper concentrations remained relatively high near the action level for the majority of the test. Anion exchange filtration resulted in cuprosolvency consistently below the AL after 27 weeks of treatment, with concentrations as low as 0.5 mg/L. Similar to pH treatment, increases in cuprosolvency of 1.5–2.1 mg/L and 0.5–0.7 mg/L, respectively, were observed for both reverse osmosis and anion exchange treated waters when treatment was suspended. GAC filtration to remove natural organic matter (NOM) was the least effective treatment, exhibiting no apparent effect on cuprosolvency in Resident B’s water.

## 4 Conclusions

Gaps in the LCR leave many residents at risk of elevated copper in their drinking water.^1,2,6,8,12,14,15^ These case studies illustrate how a utility can be in compliance with the LCR, but their water was aggressive to new copper plumbing, causing elevated cuprosolvency for a prolonged period of time. Even though there are multiple reports of elevated cuprosolvency, including for community partners and other residences with elevated copper for 5–10 years following installation of new pipes, the public utility is not required to address these problems under the LCR.^28^

It would be ideal for local authorities (e.g., utilities, health departments, cooperative extension programs) to take the lead in developing treatment and intervention guidance based on their specific water sources. In this case, the guidance herein could serve as a starting point, with the local authority adapting it to account for local considerations, acting as a centralized and reliable information source to help affected residents navigate cuprosolvency problems. Doing so would leverage in-house utility expertise with specialized knowledge of local conditions and experiences on efficacy of treatments. At a minimum, local authorities could serve as a clearinghouse, compiling experiences specific to their water and guiding residents toward effective treatments and away from those which would waste money and effort. For example, utilities with high hardness water known to cause scaling may advise residents to avoid raising pH, which could clog pipes and water heaters.^18^ Such advice may become increasingly important, given that lead service line replacements required under the Lead and Copper Rule Improvements, may generate millions of homes with new copper service lines across the country.^37^

If local authorities do not offer such assistance, residents are left to address cuprosolvency issues. This puts a large burden on consumers to develop their own expertise and conduct costly trial and error testing, repeatedly “re-inventing the wheel” of what works in that water supply, if they manage to find solutions. In other cases, some private vendors might take advantage of vulnerable residents by recommending costly interventions that are sometimes completely ineffective. While individual experiences may vary, this study documents the extraordinary efforts some residents take to address cuprosolvency issues at considerable personal and financial cost (Table 2). These two residents spent thousands of dollars ($3,800 to $30,700) and months to years of effort, investigating and attempting to solve their cuprosolvency problems. We spent nearly $75,000, in EPA grants and other funding, to assist these two residents in their search for solutions. The residents described the stress and mental/emotional toll as being the worst part of their experience. If utilities will not alleviate this burden by lending their expertise to assist residents, they also should avoid contributing to it by falsely blaming cuprosolvency on improper grounding or withholding important information. For instance, this utility could have shared information with Resident B about intermittent use of the nearby well, which clearly contributed to the variations in incoming pH and sporadic cuprosolvency problems observed in the home.

**Table 2:** Resident Costs and Implications^a^

		**Resident A**	**estimated cost**	**Resident B**	**estimated cost**
**interventions**	**point of use**	reverse osmosis, bottled water	$2,000	reverse osmosis, bottled water	$800
	**whole house *implemented***	grounding rod, dielectric union, sacrificial anode	$6,000	soda ash feed to increase pH, reagents	$1200
	**whole house *considered***	PVC insulation for copper pipe, pipe replacement with PVC	$20,000	ion exchange, GAC treatment	$1000 to $3000
**additional costs**	**testing**	meter to measure current, private laboratory tests, field colorimeter	$700	private laboratory tests, field colorimeter	$800
	**veterinary bills**	diagnosis and treatments	$22,000	diagnosis and treatments	$1000
**other impacts**	**time**	>1 year of dedicated time		∼4 months of dedicated time	
	**stress**	major toll on mental health due to so much effort leading to no changes and feeling like no one cared or would help		huge amount	
		felt horrible, gaslit, stonewalled, threatened, betrayed, ignored, helpless, hopeless, and like this experience took part of their soul			

a Values were estimated by community partners and may vary based on factors specific to each individual. Residents already had point of use reverse osmosis (RO) systems, but additional costs would apply for residents choosing to install such systems.

Although our study developed draft guidance to help address these concerns, we demonstrated that determining appropriate interventions may require case-by-case or community specific considerations after considerable effort. For example, it is now known that conventional flushing (≤2 min) can sometimes be inadequate to minimize exposure from service lines (lead),^36^ and this study similarly demonstrates that even 5 min of flushing was not sufficient to reduce copper below the AL in one home. Home plumbing configurations and residential water use concerns, particularly in water stressed regions, need to be considered to develop appropriate flushing guidance.

Finally, this study illustrated several challenges in developing and implementing guidance. We had hoped that short-term water treatments would be effective at facilitating the formation of a protective scale, as was reported in laboratory testing for GAC removal of NOM.^6^ However, our results demonstrate that long-term interventions (>36 weeks) may be necessary to control cuprosolvency for all treatments tested. Indeed, some CCTs, such as orthophosphate addition, must be continued indefinitely to maintain the benefit, putting an ongoing burden on residents to maintain the system and provide reagents.^6,38^ Further research is needed to evaluate at-home treatment strategies and consider their cost effectiveness under a range of scenarios and consumer preferences.

## Supporting Information Available

Comparisons of in-home and pipe segment cuprosolvency results for Resident A; depictions of the general categories of at-home test kit used; and a timeline for Resident B (PDF)
