# Supplementary material for: Guidance for Residents Addressing Copper Problems in Drinking Water: Opportunities and Challenges
Source: ACS ES T Water. 2024 Aug 12;4(9):4175–83. doi: 10.1021/acsestwater.4c00447 (PMC11406526; doi:10.1021/acsestwater.4c00447)
Supplement: Supplementary file 1 — ew4c00447_si_001.pdf [file ew4c00447_si_001.pdf]

Supplemental Information for "Guidance for Residents Addressing Copper Problems in Drinking Water: Opportunities and Challenges"

Rebecca Kriss,[1] Marc A. Edwards[1*]

[1]Virginia Tech, Civil and Environmental Engineering, 418 Durham Hall, Blacksburg, VA 24061

*Corresponding author: Marc A. Edwards: edwardsm@vt.edu; Address: 407 Durham Hall 1145 Perry Street, Blacksburg, VA 24060

Description of Supporting Information (2 pages, 1 Table, 1 Figure)

Table S1. Comparison of in-home sampling and pipe segment cuprosolvency results from the home of Resident A

Figure S1. Three general categories of at-home test kit utilized

Figure S2. Resident B timeline

Table S1. Comparison of in-home sampling and pipe segment cuprosolvency results from the home of Resident A

			Home Plumbing		Pipe Segments	
**Location**	**Time**	**Sample Type**	**Cu (mg/L)**	**Current (Microamps)**	**Avg. Cu (mg/L)**	**Std. Dev.**
Kitchen	9:00 PM	Flushed (5 min)	0.2			
	7:00 AM	1st Draw	0.5	103	1.1	0.1
	7:00 AM	20 sec. flush	0.9	103		
Hose Bib	9:00 PM	Flushed (1 min)	0.1			
	7:00 AM	1st Draw	0.9	98	1.1	0.0
	7:00 AM	20 sec. flush	1.0	98		
Nextdoor	9:00 PM	Flushed (1 min)	0.1			
	7:00 AM	1st Draw	0.0	135		
	7:00 AM	20 sec. flush	0.1	135		

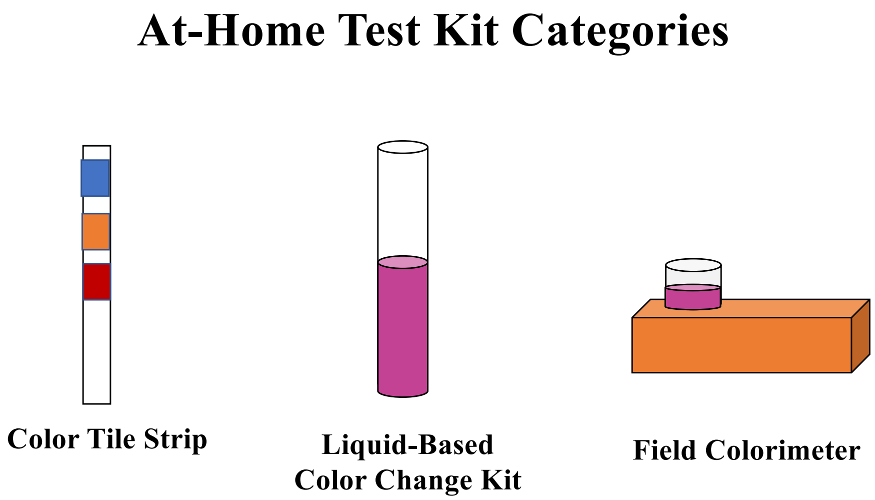

Figure S1: Three general categories of at-home test kit utilized
All tests utilize color changes to indicate copper concentrations or pH values. Values are determined by comparing the color tile or liquid color to a color key provided by the manufacturer, or via a digital readout from the field colorimeter.

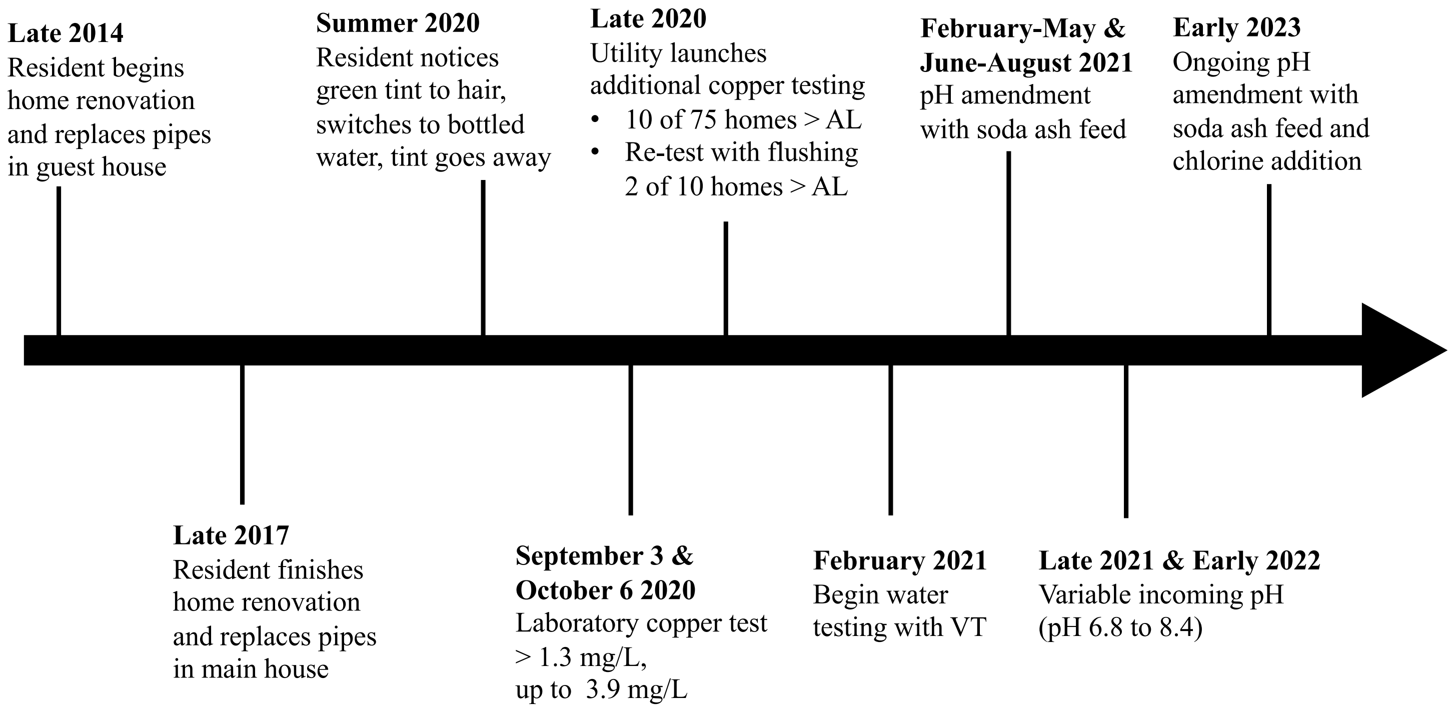

Figure S2: Resident B timeline
Timeline detailing pertinent events related to Resident B's installation of new copper pipes, determination of, and addressing cuprosolvency problems in their water.
